# Use of an artificial intelligence‐based rule extraction approach to predict an emergency cesarean section

**DOI:** 10.1002/ijgo.13888

**Published:** 2021-09-06

**Authors:** Yoko Nagayasu, Daisuke Fujita, Masahide Ohmichi, Yoichi Hayashi

**Affiliations:** ^1^ Department of Obstetrics and Gynecology Osaka Medical College Takatsuki Japan; ^2^ Department of Computer Science Meiji University Kawasaki Japan

**Keywords:** artificial intelligence, delivery, emergency cesarean section, predictive decision system, rule extraction

## Abstract

**Objective:**

One of the major problems with artificial intelligence (AI) is that it is generally known as a “black box”. Therefore, the present study aimed to construct an emergency cesarean section (CS) prediction system using an AI‐based rule extraction approach as a “white box” to detect the cause for the emergency CS.

**Methods:**

Data were collected from all perinatal records of all delivery outcomes at Osaka Medical College between December 2014 and July 2019. We identified the delivery method for all deliveries after 36 gestational weeks as either (1) vaginal delivery or scheduled CS, or (2) emergency CS. From among these, we selected 52 risk factors to feed into an AI‐based rule extraction algorithm to extract rules to predict an emergency CS.

**Results:**

We identified 1513 singleton deliveries (1285 [84.9%] vaginal deliveries, 228 emergency CS [15.1%]) and extracted 15 rules. We achieved an average accuracy of 81.90% using five‐fold cross‐validation and an area under the receiving operating characteristic curve of 71.46%.

**Conclusion:**

To our knowledge, this is the first study to use interpretable AI‐based rule extraction technology to predict an emergency CS. This system appears to be useful for identifying hidden factors for emergency CS.

## Abstract 2

This is the first study to construct a prediction system for an emergency cesarean section using an artificial intelligence‐based “white box” rule extraction approach.

## 1 INTRODUCTION

In 1985, the World Health Organization reported that the ideal cesarean section (CS) rate was 10%–15%.^1^ However, since then, CS rates have been steadily increasing around the world. In Japan, CS rates have also increased, reaching 19.2% in 2017.^2^ Compared with elective CS, emergency CS are generally known to be associated with higher risks of maternal and neonatal complications.^3^ Therefore, being able to predict the need and clarify the reasons for an emergency CS is an urgent task. In the present study, we incorporated “explainable artificial intelligence (AI)” (i.e. “white box” AI) to predict the need for an emergency CS.

Since the term was first proposed in 1956, AI has evolved. Neural networks (NN) were created as a mechanism that imitated the human brain, and these became a precursor to the spread of AI. In 2009, Housseini et al.^4^ compared predictions for CS term deliveries in nulliparas using an NN and two logistic regression models. They used samples from after 36 weeks of pregnancy. The NN showed slightly better predictive accuracy for an emergency CS than did the logistic regression models. At present, AI research has started to apply machine learning, a subfield of AI in which algorithms are trained to perform tasks by learning patterns from data as opposed to explicit rules, to deal with more complex problems than do traditional multivariate analyses. NN can also make predictions based on large amounts of patient data by learning their own relevance.^5^

However, why NN achieve better predictive accuracy for emergency CS remains unclear; this is the so‐called “black box” problem that has recently been pointed out as a disadvantage of AI, because almost all of the state‐of‐the art “black box” machine‐learning algorithms previously tested have failed to generalize well.^6^ Furthermore, the errors and biases of some NN‐based machine‐learning algorithms are difficult to understand, which is why conventional AI is often referred to as a “black box”.^5^

Rule extraction is a technique for resolving the “black box” problem by attempting to find a compromise between requirements in the following ways. The difference between the present and traditional predictive models (which doctors cannot understand and cannot explain why) is that we aim to build “explainable AI” using simple rule sets that mimic how complex predictive models make decisions for doctors and clinicians. We previously proposed the continuous recursive rule extraction (Re‐RX) algorithm with J48graft as a promising tool for rule extraction.^7^ Re‐RX with J48graft can simultaneously increase the accuracy and interpretability of extracted classification rules.

Rule extraction^8^ is a newer branch of machine learning that uses AI and focuses on why the entire data set is classified into each class. In rule extraction, the rules are typically expressed as the most popular and comprehensible symbolic descriptions as follows: “*if* (conditions 1) & (conditions 2), … & (condition *n*), *then* (target class).” Rule extraction algorithms in the medical field require a sufficient number of cases and associated final diagnoses as a supervised signal (e.g. emergency or elective CS).

The objectives of the present study were to construct a prediction system for an emergency CS using “white box” AI by explaining the reason for the CS using interpretable rules and clarifying the decision process, and to propose a new perinatal system by integrating AI with perinatal medicine.

## 2 MATERIALS AND METHODS

### 2.1 Data source

**FIGURE 1:**
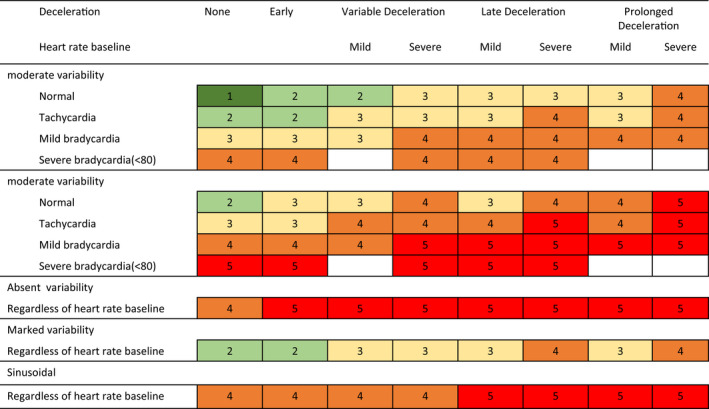
Risk levels of the five‐tier classification system as defined by the Japanese Society of Obstetrics and Gynecology. 1, Level 1 Normal pattern; 2, Level 2 Benign variant pattern; 3, Level 3 Mild variant pattern; 4, Level 4 Moderate variant pattern; 5, Level 5 Severe variant pattern [Colour figure can be viewed at wileyonlinelibrary.com]

Data were collected from all perinatal records of all delivery outcomes at Osaka Medical College between December 2014 and July 2019. The present study was approved by our institutional review board (No. 2831). Consent was not required because of the retrospective study design. We identified the delivery method for all deliveries after 36 weeks of gestation as either (1) vaginal delivery or elective CS, or (2) emergency CS. We studied cases after 36 weeks of pregnancy because our institution has an open system for pregnant women after 34–36 weeks of pregnancy. The reasons for the emergency CS were when labor began before the elective CS, placental abruption, failure to induce labor after premature rupture of the membranes or exceeding the estimated due date, prolapse of the umbilical cord, uterine rupture, or non‐reassuring fetal status based on cardiotocography. The Japan Society of Obstetrics and Gynecology has defined abnormalities in a fetal heart monitor as levels 1–5.^9^ We determined the non‐reassuring fetal status to be the continuation of level 3 or the appearance of level 4 and level 5 according to the Japan Society of Obstetrics and Gynecology guidelines (Figure 1).^10^

### 2.2 Variable selection

Several intricately intertwined risk factors (RF) are known for emergency CS.^11^, ^12^, ^13^ In the present study, we considered RF and selected 52 variables associated with the prediction of an emergency CS (Tables 1 and 2). Among these 52 variables was body mass index (calculated as weight in kilograms divided by the square of height in meters). Gestational hypertension and pre‐eclampsia were both defined as blood pressure >140/90 mm Hg, pre‐eclampsia was also defined as urinary protein/creatinine >0.27. Gestational diabetes was diagnosed based on a 75‐g oral glucose tolerance test (fasting blood glucose >92 mg/dL, 1‐h blood glucose >180 mg/dL, or 2‐h blood glucose 153 mg/dL). Preterm birth was defined as a birth before 37 weeks of pregnancy. Fetal growth restriction was diagnosed in utero with an estimated fetal weight <1.5 standard deviations from the Japanese standard. Placenta previa was defined as a placenta covering the internal uterine ostium.

**TABLE 1:** Maternal characteristics and complications^a^

	(1) Vaginal delivery or elective CS	(2) Emergency CS	*P* value
Maternal age, years	33.0 (27.0–40.5)	35.0 (28.0–41.5)	N.S.
Weight, kg	52.1 (41.1–63.1)	53.0 (42.0–64.0)	N.S.
Height, cm	159.0 (151.0–167.0)	157.1 (150.1–163.1)	< 0.01
BMI	20.5 (16.4–24.6)	21.4 (17.2–25.6)	< 0.05
Gravida	2 (0–4)	1 (0–2)	< 0.01
Parity	1 (0–3)	1 (0–2)	< 0.001
Spontaneous abortion	0 (0–2)	0 (0–2)	N.S.
Infertility treatment	228 (21.1%)	44 (22.9%)	N.S.
Smoking	41 (3.7%)	5 (2.5%)	N.S.
Alcohol consumption	26 (2.5%)	7 (4.0%)	N.S.
Family history			
Hypertension	341 (33.4%)	48 (25.8%)	< 0.05
Diabetes	242 (23.7%)	45 (24.5%)	N.S.
Maternal history			
Hyperthyroidism	8 (0.7%)	5 (2.5%)	< 0.05
Hypothyroidism	20 (1.8%)	3 (1.5%)	N.S.
Overt DM	9 (0.8%)	3 (1.5%)	N.S.
Hypertension	11 (1.0%)	2 (1.0%)	N.S.
Hepatitis	6 (0.5%)	1 (0.5%)	N.S.
Respiratory disease	23 (2.1%)	1 (0.5%)	N.S.
Autoimmune disease	32 (2.9%)	5 (2.5%)	N.S.
Collagen disease	32 (2.9%)	3 (1.5%)	N.S.
Appendicitis	24 (2.2%)	3 (1.5%)	N.S.
Central nervous system disease	17 (1.5%)	3 (1.5%)	N.S.
Mental disease	53 (4.8%)	11 (5.6%)	N.S.
Renal disease	14 (1.3%)	1 (0.5%)	N.S.
Hematologic disease	3 (0.3%)	1 (0.5%)	N.S.
Myoma	38 (3.4%)	5 (2.5%)	N.S.
Uterine operation	29 (2.6%)	7 (3.6%)	N.S.

Abbreviations: BMI, body mass index (calculated as weight in kilograms divided by the square of height in meters); CS, cesarean section; DM, diabetes mellitus; N.S., not significant.

^a^ Values are presented as mean (range) or as number (percentage).

**TABLE 2:** Obstetrics history and perinatal outcomes between two classes^a^

Obstetrics history	(1) Vaginal delivery or elective CS	(2) Emergency CS	*P* value
Cesarean section	0 (0–2)	1 (0–3)	<0.001
Gestational hypertension	11 (1.0%)	2 (1.0%)	N.S.
Pre‐eclampsia	17 (1.5%)	2 (1.0%)	N.S.
Preterm birth	0 (0–3)	0 (0–2)	N.S.
Premature labor	43 (3.9%)	2 (1.0%)	<0.05
Cervical laceration	4 (0.4%)	2 (1.0%)	N.S.
Placenta abruption	4 (0.4%)	2 (1.0%)	N.S.
Infection	10 (0.9%)	4 (2.0%)	N.S.
Gestational diabetes mellitus	23 (2.1%)	4 (2.0%)	N.S.
Gestational age, weeks	38.3 (36.0–39.4)	39.3 (36.2–39.6)	N.S.
CS	263 (23.8%)	197 (15.1%)	—
Emergency CS	—	197 (15.1%)	—
Vaginal delivery	842 (76.2%)	—	—
Vacuum delivery	131 (15.6%)	—	—
Augmentation of labor	135 (12.2%)	27 (13.7%)	N.S.
Pregnancy complications			
Fetal growth restriction	30 (2.7%)	19 (9.6%)	<0.0001
Induction of labor	159 (14.4%)	37 (18.8%)	N.S.
Hypertensive disorders of pregnancy	60 (5.4%)	22 (11.2%)	<0.001
Stillbirth	6 (0.5%)	0 (0%)	—
TORCH syndrome	2 (0.2%)	(0%)	—
Gestational diabetes mellitus	24 (2.2%)	2 (1.0%)	N.S.
Premature rupture of membranes	153 (13.8%)	60 (30.5%)	<0.001
Placenta previa	17 (1.5%)	4 (2.0%)	N.S.
Single umbilical artery	3 (0.3%)	1 (0.5%)	N.S.
Abnormal umbilical cord insertion (marginal or velamentous insertions)	60 (6.3%)	17 (8.6%)	N.S.
Deep vein thrombosis	2 (0.2%)	1 (0.5%)	N.S.
Neonatal birth weight (g)	2974 (2437–3511)	2894 (2156–3632)	<0.01

Abbreviations: CS, cesarean section; N.S., not significant; TORCH, (T)oxoplasmosis, (O)ther Agents, (R)ubella, (C)ytomegalovirus, and (H)erpes simplex.

^a^ Values are presented as mean (range) or as number (percentage).

### 2.3 Artificial intelligence‐based rule extraction technology

The Re‐RX algorithm recently developed by Setiono et al.^14^ was originally designed as a rule extraction tool. The Re‐RX algorithm enables a hierarchical, recursive consideration of discrete variables (variables that have only one integer value within a range of values, e.g. male [1]/female [0], category [0,1,2,3]) before the analysis of continuous data (variables that can have any value within a defined range, e.g. 0.123, 45.67). It therefore provides a rule extraction method that offers both accuracy and interpretability because of its ability to discriminate between discrete and continuous attributes in the antecedent (*if* condition) of each extracted rule. The Re‐RX algorithm eliminates continuous attributes before the C4.5 decision tree^15^ is generated using only discrete attributes.

The Re‐RX algorithm is a “white box” (more understandable) model that can provide highly accurate and concise classification and be easily explained and interpreted in accordance with the concise extracted rules associated with *if–then* forms; therefore, this type of model is often preferred by physicians and clinicians. The Re‐RX algorithm provides an accurate rule extraction method that also offers comprehensibility by generating perfect separation between discrete and continuous attributes in the antecedent (*if* condition) of each extracted rule. The outline of the Re‐RX algorithm is as follows^16^:

#### 2.3.1 Re‐RX algorithm (*S,D,C*)

Input: A set of data samples *S* with discrete attributes *D* and continuous attributes *C*.

Output: A set of classification rules.

1. Train and prune an NN using data set *S* and all its *D* and *C* attributes.
2. Let *Dʹ* and *Cʹ* be the sets of discrete and continuous attributes in *Sʹ*, respectively, still present in the network, and let *Sʹ* be the set of data samples correctly classified by the pruned network.
3. If *Dʹ* = φ, then generate a hyperplane, $\sum_{C_{i}\in C^{\prime }}W_{i}C_{i}=W_{0}$, that separates the two groups of samples. Use the constant *W*_0_ and the rest of the coefficients *W_i_* of the hyperplane to split the samples into *Sʹ* according to the values of the continuous attributes *Cʹ*, and then stop. Otherwise, use only the discrete attributes *Dʹ* to generate the set of classification rules *R* for data set *Sʹ*.
4. For each rule, *R_i_* is generated:

If support (*R_i_*) > *δ*_1_ (covering rate) and error (*R_i_*) > *δ*_2_ (error rate), then.

- Let *S_i_* be the set of data samples that satisfy the condition of rule *R_i_* and let *D_i_* be the set of discrete attributes that do not appear in rule condition *R_i_*.
- If *D_i_* = φ, then generate a hyperplane to split the samples in *S*_i_ according to the values of their continuous attributes *C*_i_, and then stop.
- Otherwise, call Re‐RX (*S_i_*,*D_i_*,*C_i_*).

**FIGURE 2:**
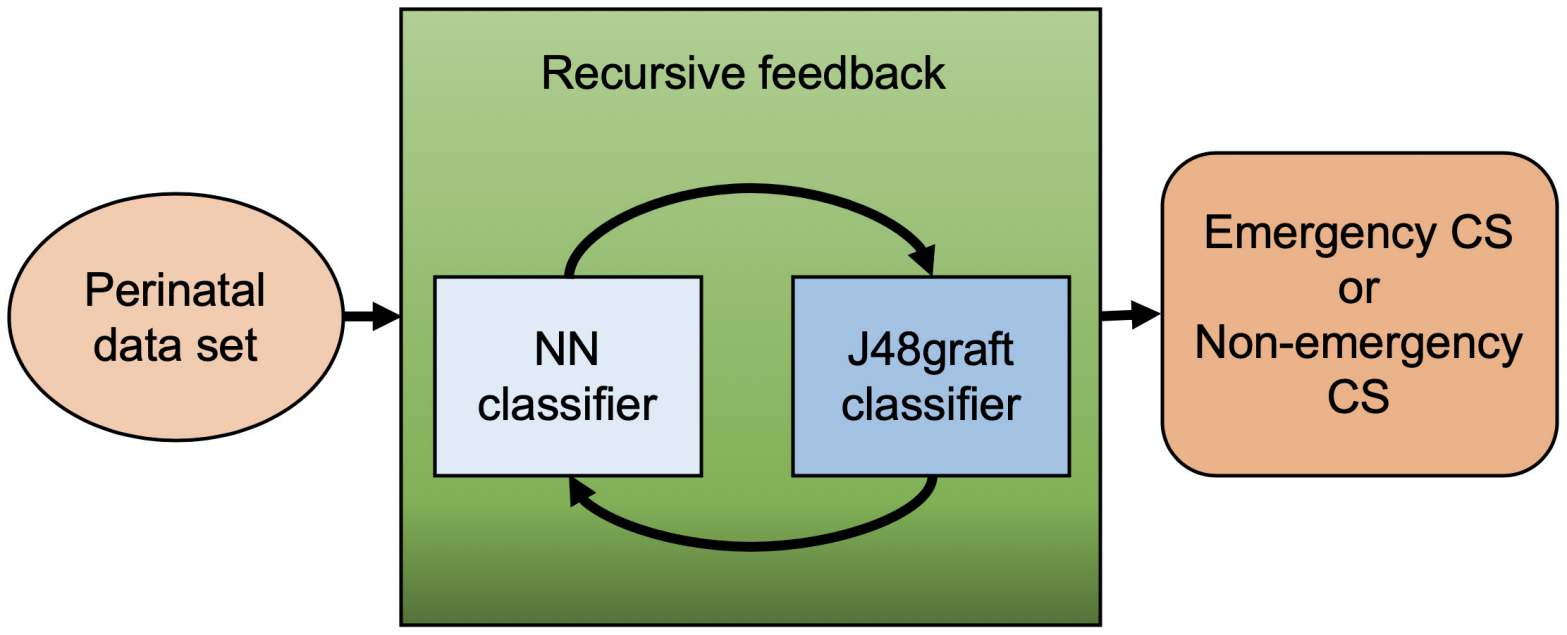
Schematic overview of Re‐RX with J48graft. Abbreviations: CS, cesarean section; NN, neural network [Colour figure can be viewed at wileyonlinelibrary.com]

The Re‐RX algorithm prioritizes the extraction of rules comprising discrete attributes, while continuous Re‐RX with J48graft belongs to the accuracy–priority type and uses both discrete and continuous attributes to generate the J48graft decision tree, which results in slightly increased complexity. With mostly continuous input features (variables), we prioritize selecting and expressing important/influential continuous features in perinatal data sets that are expressed in an *if–then* form in J48graft. For a better understanding of the mechanism underlying continuous Re‐RX with J48graft,^16^ a schematic overview is provided in Figure 2.

### 2.4 Performance measures

**FIGURE 3:**
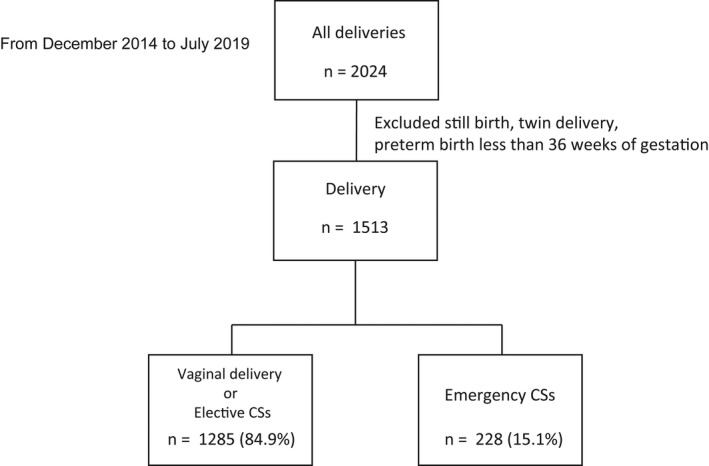
CONSORT flowchart

To guarantee the validity of the results and evaluate the classification rule accuracy of the test data sets, we performed five‐fold cross‐validation, which is a technique used to assess how a classifier performs when classifying new instances of a task (Figure 3). We trained the perinatal data set using Re‐RX with J48graft and obtained 10 runs of five‐fold cross‐validation for the training accuracy, test accuracy, average number of extracted rules, and area under the receiving operating characteristic curve on a conventional personal computer (Intel Core i7 7500 U; 2.7 GHz; 8 GB RAM).

### 2.5 Statistical analysis

To compare clinical parameters between the patients who underwent a CS, we used a *t* test (*P* < 0.05 was considered significant) for comparisons. Data were analyzed using SPSS software (Mac version 20.0 J; IBM).

## 3 RESULTS

### 3.1 Statistical comparisons between the emergency and non‐emergency CS classes

In total, we identified 1513 cases of singleton deliveries after 36 weeks of pregnancy at Osaka Medical College between December 2014 and July 2019, among which, 1285 (84.9%) were vaginal deliveries or elective CS and 228 (15.1%) were emergency CS.

Table 1 shows the maternal characteristics for all cases. In a univariate analysis, differences were found between the (1) vaginal delivery or elective CS (non‐emergency CS) and (2) emergency CS classes. Table 2 shows the differences in obstetrics history and perinatal outcomes between two classes. After multiple regression, significant differences were found in parity, height, hyperthyroidism, history of CS, premature membrane rupture, and fetal growth restriction (Table 3).

**TABLE 3:** Results of the multiple logistic regression analysis

Variable	Adjusted relative risk (95% CI)	*P* value
Parity	0.457	0.333–0.626
Maternal height	0.956	0.922–0.991
Hyperthyroidism	6.055	1.380–26.569
History of cesarean section	3.042	2.137–4.329
Premature membrane rupture	3.372	2.128–5.246
Fetal growth restriction	8.635	3.529–21.126

Abbreviation: CI, confidence interval.

Table 4 shows the 15 concrete rules that were used to discriminate the emergency CS from the vaginal delivery or elective (non‐emergency) CS class in the perinatal data sets using the AI‐based rule extraction approach. Each condition in a rule is a conjunction.

**TABLE 4:** Fifteen concrete rules that were used to discriminate the emergency and non‐emergency CS perinatal data sets using the AI‐based rule extraction approach

Rule 1	BW ≤ 2456	CS ≤ 0.32	PB ≤ 0.13	Parity ≤ 2	BW ≤ 2118	GDM = 0					Class 1^d^
Rule 2	BW ≤ 2456	CS ≤ 0.32	PB ≤ 0.13	Parity ≤ 2	BW ≤ 2118	GDM = 0	BW ≤ 1152				Class 1
Rule 3	BW ≤ 2456	CS ≤ 0.32	PB ≤ 0.13	Parity ≤ 2	BW ≤ 2118	GDM = 0	BW ≤ 1152	Height^a^ ≤ 159.5			Class 1
Rule 4	BW ≤ 2456	CS ≤ 0.32	PB ≤ 0.13	Parity ≤ 2	BW ≤ 2118	GDM = 1	BW > 1152	Height ≤ 159.5	HDP = 0	Age ≤ 29.5	Class 2^e^
Rule 5	BW ≤ 2456	CS ≤ 0.32	PB ≤ 0.13	Parity ≤ 2	BW ≤ 2118	GDM = 1	BW > 1152	Height ≤ 159.5	HDP = 0	Age > 29.5	Class 1
Rule 6	BW ≤ 2456	CS ≤ 0.32	PB ≤ 0.13	Parity ≤ 2	BW ≤ 2118	GDM = 1	BW > 1152		HDP = 0		Class 1
Rule 7	BW ≤ 2456	CS ≤ 0.32	PB ≤ 0.13	Parity ≤ 2	BW > 2118	Previa^b^ = 0	Fertility treatment = 0				Class 2
Rule 8	BW ≤ 2456	CS ≤ 0.32	PB ≤ 0.13	Parity ≤ 2	BW > 2118	Previa = 0	Fertility treatment = 1	Age^c^ ≤ 33	BMI ≤ 21.4		Class 2
Rule 9	BW ≤ 2456	CS ≤ 0.32	PB ≤ 0.13	Parity ≤ 2	BW > 2118	Previa = 0	Fertility treatment = 1	Age ≤ 33	BMI > 21.4		Class 1
Rule 10	BW ≤ 2456	CS ≤ 0.32	PB ≤ 0.13	Parity ≤ 2	BW > 2118	Previa = 0	Fertility treatment = 1	Age ≤ 33	BMI > 21.4		Class 2
Rule 11	BW ≤ 2456	CS ≤ 0.32	PB ≤ 0.13	Parity ≤ 2	BW > 2118	Previa = 1					Class 1
Rule 12	BW ≤ 2456	CS ≤ 0.32	PB ≤ 0.13	Parity > 2							Class 2
Rule 13	BW ≤ 2456	CS ≤ 0.32	PB > 0.13								Class 2
Rule 14	BW ≤ 2456	CS > 0.32									Class 1
Rule 15	BW > 2456										Class 2

Abbreviations: AI, artificial intelligence; BMI, body mass index (calculated as weight in kilograms divided by the square of height in meters); BW, birth weight; CS, cesarean section; GDM, gestational diabetes mellitus; HDP, hypertensive disorders of pregnancy; PB, preterm birth.

^a^ Maternal height.

^b^ Placenta previa.

^c^ Maternal age.

^d^ Emergency CS.

^e^ Non‐emergency CS.

### 3.2 Performance of the continuous Re‐RX algorithm with J48graft

We achieved a test accuracy of 81.90% and an area under the receiving operating characteristic curve of 71.46 for the perinatal data set. This means that the performance of the predictive model to discriminate the emergency CS from the non‐emergency CS class was relatively high. The predictive model consisted of the entire set of 15 extracted rules. It took about 3 s to train the data set using continuous Re‐RX with J48graft. The testing time was negligible.

### 3.3 Extracted rules

#### 3.3.1 Rules 1 and 12–15

Rule 1 included low birth weight (≤2118 g), a history of CS, a history of preterm birth, parity, and no gestational diabetes mellitus. It is known that late‐onset fetal growth restriction is an RF in short‐ and long‐term prognoses.^17^ Moreover, it is well known that the first delivery is a most frequent cause of an emergency CS.^18^

#### 3.3.2 Rules 2 and 3

Rules 2 and 3 included very low birth weight (≤1152 g), which can cause non‐reassuring fetal status more easily than can low birth weight. In rule 3, maternal height ≤159.5 cm was included.

#### 3.3.3 Rules 4–6

Rules 4 and 5 included maternal age (≤29.5 or >29.5 years). Moreover, GDM was an RF for an emergency CS in rules 4–6.

#### 3.3.4 Rule 7

Rule 7 included birth weight ≤2456 g and >2118 g when there is no placenta previa, no fertility treatment, nullipara, and no history of CS or preterm birth.

#### 3.3.5 Rules 8–10

Rules 8–10 included maternal body mass index (≤21.4 or >21.4) and fertility treatment.

#### 3.3.6 Rule 11

Rule 11 included placenta previa. When pregnant women have vaginal bleeding, an emergency CS is frequently performed.

#### 3.3.7 Rule 12

Higher parity increases the chance of a vaginal delivery.

## 4 DISCUSSION

An emergency CS increases the risk of maternal complications and infant mortality.^19^, ^20^ In the present study, we included 52 extracted factors in J48graft for the prediction of an emergency CS, resulting in 15 rule sets, which included 12 factors. These factors are well‐known RF for an emergency CS.^4^, ^21^ Regarding the birth weight factor, there were three cut‐off points: 2456, 2118, and 1152 g. It is also well known that a low birth weight (<2500 g) can easily cause an emergency CS.^22^ We performed univariate analysis on each factor. As a result, the 10 factors that appeared in the rule sets were significantly different in the emergency CS group. Therefore, we considered that these rule sets could approximate the predictive model.

To date, several emergency CS prediction systems have been reported, many of which have used multivariate analysis. Whereas multivariate analysis identified individual causal factors for an emergency CS, in the present study, the coefficient for each risk was presented in the form of rules.

In 2017, Papoutsis et al.^23^ proposed a risk assessment tool for the prediction of an emergency CS and reported several RF using a scoring model. That scoring model has the advantage of being able to calculate a large number of factors and their values, but it cannot have several cut‐offs for one RF.

Furthermore, in 2018, to identify RF for an emergency CS, Campillo‐Artero et al.^21^ used likelihood ratios and logistic regression, and fitted a conditional inference tree (CTREE). The decision tree is a major competitor of classification and regression trees given an identified bias of the latter toward numeric and categorical variables with many categories. Consequently, CTREE was found to improve predictions compared with classification and regression trees. However, the CTREE algorithm assumes independence between samples and is more likely to select a split in correlated data. In multilevel data sets, this can lead to complex trees that may overfit the training data.

In 2020, a systematic review reported that the machine‐learning algorithms were superior to logistic regression for predicting prenatal outcomes, including a CS.^24^ According to that review, gradient boosting, decision trees, and RF were significantly better than the logistic regression for predicting a CS. They then recommended a re‐analysis of existing logistic regression models through a comparison with machine‐learning algorithms.

To the best of our knowledge, only one algorithm has been proposed to extract rules from decision tree ensembles such as random forest models.^21^ That algorithm did not fully outperform the accuracy and interpretability (e.g. the number of rules extracted) obtained by continuous Re‐RX with J48graft. The main strength of the present study is that it explains the reason why an emergency CS is required by using interpretable and concise rules derived through continuous Re‐RX with J48graft. Second, numerous RF (52 variables in the present study) and their cut‐off values can be obtained at the same time by using the proposed algorithm. Therefore, it is possible to investigate emergency CS clearly and in a timely fashion using a unique mechanism consisting of NN and C4.5 decision trees.

Artificial intelligence is still often seen as a “black box”, which makes it difficult for obstetricians to accept, because the reasons for decisions are not clearly explained. Moreover, the weak points of “black box” AI to detect errors and biases have been pointed out.^5^ A review article about clinical safety in AI reported that predictions by rules‐based systems can be explained in terms of inputs. The AI‐based rule extraction approach used in that study can explain why an emergency CS is performed.^7^ The emergency CS prediction system using continuous Re‐RX with J48graft described in the present study explains risk based on a rule set that discriminates between emergency and non‐emergency CS. As a result, the system can be considered “white box” AI for perinatal medicine. Furthermore, the support of AI could be expected to improve the perinatal prognosis. One of the present authors previously proposed a non‐invasive prediction method for non‐alcoholic steatohepatitis in Japanese patients with morbid obesity using this algorithm.^25^ That work included a discussion about extracted rules and RF.

The present study has several limitations. First, we extracted 52 RF, but we did not include other RF, such as the sex and maternal race of the newborn. The reason why we chose not to include these factors is that most of the data are from Japan, and we judged that they were not important. Second, the data set was from one hospital, which might introduce some degree of bias in the data set. More cases need to be considered to construct a model with better predictive ability. Third, to enable comparisons with previous studies,^3^, ^4^ we constructed a prediction system that included planned vaginal deliveries and planned CS. However, a predictive system for emergency CS after a vaginal delivery would also be required.

To the best of our knowledge, this is the first study to use “white box” AI‐based rule extraction technology to predict the need for an emergency CS. This system extracts accurate and concise rules to identify multiple RF in the field of labor and appears useful for identifying RF for an emergency CS, which could improve perinatal results in the future.

## CONFLICTS OF INTEREST

The authors have no conflicts of interest.

## AUTHOR CONTRIBUTIONS

YN contributed to the collection and interpretation of the data. DF contributed to the collection of the data. MO contributed to the interpretation of the data. YH contributed to the analysis of the data. YN and YH contributed to the conception and design of the study, and to the writing and revision of the manuscript.
